# Data-Driven Twisted String Actuation for Lightweight and Compliant Anthropomorphic Dexterous Hands

**DOI:** 10.3390/biomimetics10090621

**Published:** 2025-09-15

**Authors:** Zhiyao Zheng, Jingwei Zhan, Zhaochun Li, Yucheng Wang, Chanchan Xu, Xiaojie Wang

**Affiliations:** 1 College of Mechanical and Electronic Engineering, Nanjing Forestry University, Nanjing 210037, China; zzybailixi@njfu.edu.cn; 2 Institute of Intelligent Machines, Hefei Institutes of Physical Science, Chinese Academy of Sciences, Hefei 230031, Chinaxjwang@iamt.ac.cn (X.W.); 3 University of Science and Technology of China, Hefei 230031, China; 4 School of Artificial Intelligence, Anhui Polytechnic University, Wuhu 241000, China

**Keywords:** twisted string actuator, anthropomorphic dexterous hand, data-driven modelling, back propagation neural network, variable-load control, biomimetic design

## Abstract

Anthropomorphic dexterous hands are crucial for robotic interaction in unstructured environments, yet their performance is often constrained by traditional actuation systems, which suffer from excessive weight, complexity, and limited compliance. Twisted String Actuators (TSAs) offer a promising alternative due to their high transmission ratio, lightweight design, and inherent compliance. However, their strong nonlinearity under variable loads poses significant challenges for high-precision control. This study presents an integrated approach combining data-driven modeling and biomimetic mechanism innovation to overcome these limitations. First, a data-driven modeling approach based on a dual hidden-layer Back Propagation Neural Network (BPNN) is proposed to predict TSA displacement under variable loads (0.1–4.2 kg) with high accuracy. Second, a lightweight, underactuated five-finger dexterous hand is developed, featuring a biomimetic three-phalanx structure and a tendon-spring transmission mechanism, achieving an ultra-lightweight design. Finally, a comprehensive experimental platform validates the system’s performance, demonstrating precise bending angle prediction (via integrated BPNN–kinematic modeling), versatile gesture replication, and robust grasping capabilities (with a maximum fingertip force of 7.4 N). This work not only advances TSA modeling for variable-load applications but also provides a new paradigm for designing high-performance, lightweight dexterous hands in robotics.

## 1. Introduction

Anthropomorphic dexterous hands serve as the core end effectors for robot interaction with the physical environment. Their performance critically determines a robot’s operational capabilities in complex unstructured scenarios such as service tasks, medical rehabilitation, and precision assembly [1,2,3]. The actuation scheme represents a key bottleneck constraining the lightweight design, compliance, and force control precision of these hands. The driving methods of dexterous hands can mainly be divided into three types: motor-driven, fluid-driven, and smart material-driven.

Fluid-driven actuators achieve movement by compressing and releasing fluids, mainly being used in pneumatically driven soft dexterous hands [4]. This type of dexterous hand offers high flexibility and good safety, but it usually requires large compressors to generate high pressure. This results in bulky equipment and the need for supporting pipelines, limiting its application in mobile platforms and specific designs.

Furthermore, smart material-driven dexterous hands also have broad application prospects. Common smart material actuators include shape memory alloy (SMA) actuators [5], electroactive polymer (EAP) actuators [6], and supercoiled polymer (SCP) actuators [7]. EAPs utilize the inverse piezoelectric effect, boasting advantages such as high linearity, high stress, and fast response; however, their stroke is small (usually at the micrometer or nanometer level), which restricts their application in large-range motion scenarios. SCPs and SMAs drive dexterous hands through heat-induced contraction, and they offer benefits like low noise, compact structure, light weight, flexible wiring, and free mobility. In particular, SCP actuators provide a large driving range and significant output force. Nevertheless, both SMA and SCP actuators are thermally driven systems, suffering from problems of slow response speed and low energy efficiency.

Motor-driven dexterous hands are the most widely used type currently. Based on the combination of motors with other components, motor-driven dexterous hands can be further categorized into subtypes such as motor–gear combinations [8], motor–mechanical linkage combinations [9], motor–tendon combinations [10], and motor–twisted string combinations [11]. Dexterous hands with motor–gear or motor–mechanical linkage combinations feature high control accuracy and a fast response speed. However, their high structural rigidity, heavy weight, and complex mechanical structure limit the flexibility and interactivity of the dexterous hands to a certain extent. Dexterous hands driven by motor–tendon combinations can improve flexibility and dexterity; nevertheless, the need to install motors vertically along the driving direction makes practical operation in the hand or arm extremely difficult. This not only affects the compactness of the structural design but may also restrict the movement range of the wrist. In addition, the transmission efficiency between the motor and tendons is relatively low—larger motor power is required to achieve sufficient finger force, which in turn increases the size and weight of the dexterous hand. To address the aforementioned issues, dexterous hands driven by the motor–twisted string combination have emerged. This drive method requires no external mechanisms; it only converts the motor’s rotational motion into linear motion via the string, and the string can be arranged in any direction—this makes the motor–twisted string system more compact and flexible compared with the traditional motor–tendon system. Furthermore, the motor–twisted string system can output greater driving force with a smaller input torque, and its output force can exceed that of the motor–tendon system by more than five times. Therefore, this system can adopt motors with smaller power and lighter weight to achieve the same output force, further improving the efficiency and performance of the overall system.

Through the analysis of different driving methods, it can be seen that each type has its own advantages and disadvantages in the application of dexterous hands. The selection of an appropriate driving method requires comprehensively considering the functional requirements of the dexterous hand and its practical application scenarios.

**Figure 1:**
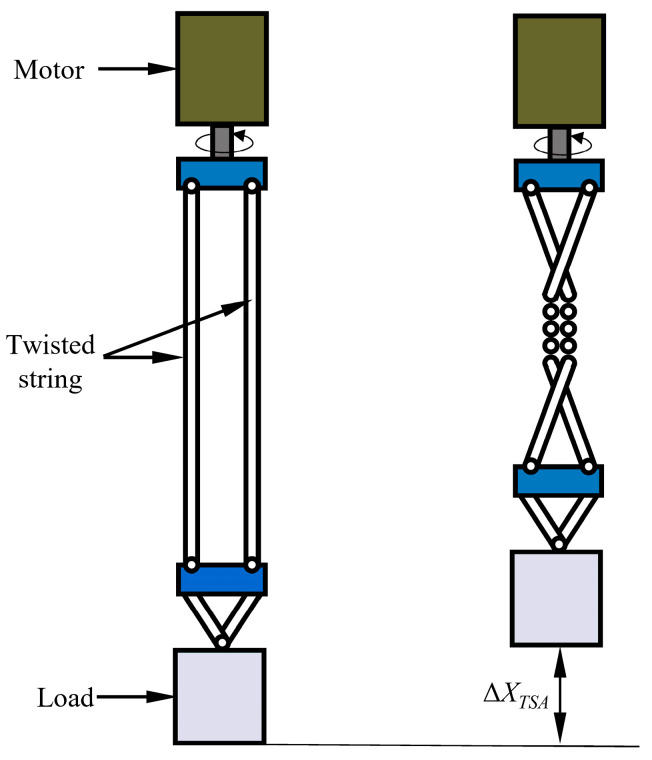
Schematic diagram of TSA.

TSAs have gained widespread attention in recent years as an emerging flexible drive technology due to their high transmission ratio, simple structure, light weight, and inherent compliance [12,13]. As illustrated in Figure 1, a TSA typically consists of a motor, a load, and one or more twisted strings. One end of the twisted string is connected to a rotating motor, while the other end is attached to the load. Under the rotation of the motor, the twisted strings either twist themselves or wrap around, shortening their length and driving the load to produce linear motion [14]. This unique mechanism allows the TSA to effectively convert the rotational motion of the motor into the linear motion of the load [15]. The TSA is particularly well-suited for dexterous hands that require high power density and compliant interaction in space-constrained environments [16,17]. For example, Sonoda et al. [15] demonstrated the advantages of TSAs in low-cost, lightweight dexterous hands. Zhang et al. [16] developed an anthropomorphic dexterous hand with an all-soft structure, which is driven by TSAs and integrated into an arm system. This system can achieve 31 out of the 33 grasping modes in the Feix GRASP taxonomy, demonstrating excellent dexterity and grasping diversity.

However, in applications requiring high-precision position or force control, the TSA still faces a major challenge: its drive process is highly nonlinear and extremely sensitive to the load. Under the same motor input, an increase in load significantly reduces the output displacement. This strong nonlinearity arises from the complex elastic deformation, radius change, friction, and geometric evolution during the twisting process. To address this challenge, researchers have developed a series of mathematical models. Based on the research of Wurtz et al. on the TSA system, Palli et al. [18] deeply explored the relationship between the twisted string stiffness and the driving performance. Ryu et al. introduced the assumption of variable twisted string radius on the basis of the traditional constant radius model, established the TSA variable radius model [19] and TSA variable stiffness model [20], and considered the influence of load, proposing a TSA variable-load mathematical model with bias [21]. Lin and Chu [22] further incorporated parameter estimation and compensation techniques. Nedelchev et al. [23] were the first to study the natural nonlinear oscillation behavior in Twisted String Actuators (TSAs). Based on the energy balance equation and dynamic equation, they established a mathematical model of the TSA and conducted an in-depth analysis of the energy retention characteristics and damped oscillation behavior of the TSA. Liu et al. [24] established forward and inverse bidirectional kinematic models of the TSA based on the Invertible Neural Network (INN), which improved the accuracy of forward and inverse kinematic modeling for the TSA. In particular, this approach reduced errors in regions such as after multiple strokes and in low-angle ranges. Konda et al. [25] considered the friction between twisted strings and incorporated the reverse torque generated by changes in twisted string curvature, thereby establishing an enhanced dynamic model of the TSA. This model improved the accuracy of predicting the dynamic behavior of TSAs driven by low-torque and low-speed motors.

Although these models (including geometric statics and their improved forms) are effective under steady-state or simplified conditions, they rely heavily on idealized assumptions, neglecting key influencing factors such as material properties and string diameter differences, and exhibit high sensitivity to load variations. As a result, existing physical models fail to provide sufficiently accurate predictions under a wide range of variable-load conditions, severely limiting the application of TSAs in high-precision dexterous hand control.

Current data-driven methods, such as neural networks, have shown great potential in handling complex nonlinear systems [26,27]. However, their application in addressing the precise modeling of TSAs under variable-load conditions has not been fully explored. Moreover, the effective integration of high-precision TSA models into dexterous hand systems, achieving validation from drive prediction to precise control of the end effector, remains a gap in current research.

To address the aforementioned challenges, this paper proposes a solution integrating data-driven modeling and biomimetic mechanism innovation. The main contributions are as follows:

1. A data-driven modeling framework: A BPNN-based approach is developed for high-precision displacement prediction of TSAs under variable loads (0.1–4.2 kg). Compared with traditional methods, the proposed data-driven approach significantly improves prediction accuracy.
2. We developed a lightweight TSA-driven anthropomorphic dexterous hand based on a biomimetic three-joint finger structure, a tendon-spring transmission system, and 3D-printed resin materials. The anthropomorphic dexterous hand (excluding the motor) weighs 335 g, with a size approximately 1.2 times that of a real human hand. Furthermore, by integrating a BPNN-based displacement model with the kinematic model of the fingers, we established a predictive model capable of more accurately estimating finger bending angles from motor rotation angles. This lays a solid foundation for the precise control of TSA-driven dexterous hands.
3. Systematic validation and performance evaluation: An experimental platform is established, comprising the TSA drive unit, the dexterous hand body, the bending angle sensor, and a real-time control system. Comprehensive experiments validate the following:
  - The accuracy of the finger’s bending angle prediction model combining the BPNN model with dexterous hand kinematics.
  - The TSA-driven dexterous hand demonstrates excellent bending capability (achieving various human-like gestures) and practical grasping performance.

This study not only provides an effective modeling tool for precise TSA control under variable-load conditions but also opens a new technical pathway for developing the next generation of lightweight, high-performance anthropomorphic dexterous hands.

## 2. TSA Displacement Characterizations

A specialized test platform was developed to characterize TSA displacement as a function of motor rotation angles and applied loads, with data acquisition performed under controlled conditions.

### 2.1. Experimental Platform Setup

**Figure 2:**
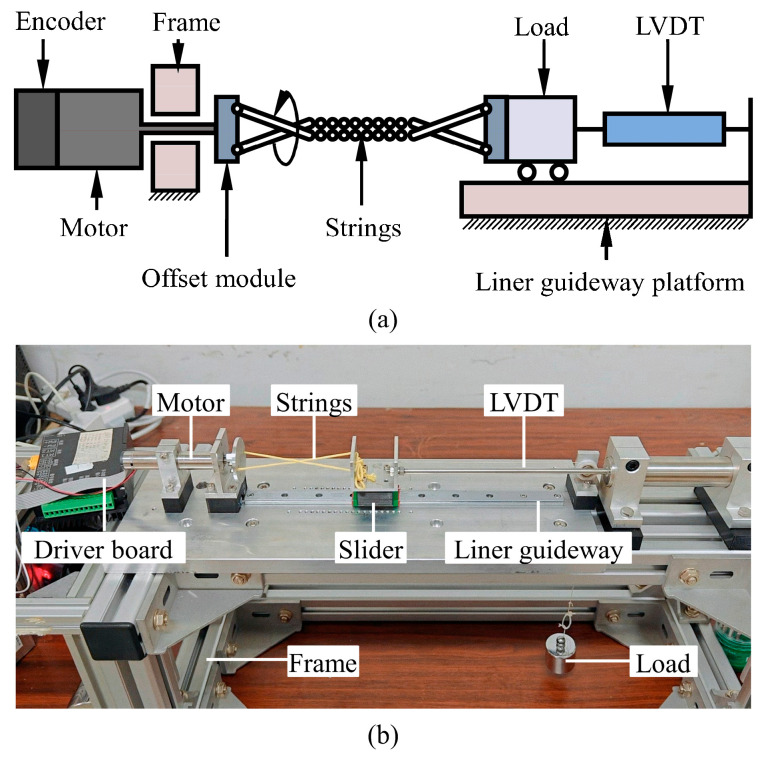
Schematic diagram (**a**) and experimental setup (**b**) for TSA displacement testing.

The TSA displacement characterization platform is depicted in Figure 2, where Figure 2a provides the schematic representation and Figure 2b shows the actual experimental setup. The setup consisted of a Direct Current (DC) motor (Maxon DCX16L) with an integrated encoder (Maxon ENX16 EASY), a suspended load, a linear guideway (Hiwin, MG series), a Linear Variable Differential Transformer (LVDT, W-DC series with a 20 cm stroke), and twisting strings (Maxon DCX16L&Maxon ENX16 EASY: DC motor and integrated encoder: maxon motor ag, Sachseln, Switzerland; Hiwin, MG series: Hiwin Technologies Corp., Taichung, Taiwan, China; Linear Variable Differential Transformer (LVDT): Beijing Tianyu Hengchuang Sensor Technology Co., Ltd., Beijing, China.)

In the specific configuration of the experimental setup, the encoder is connected to the motor and horizontally mounted on the frame. The motor output shaft is connected to the twisted string via a custom fixture, and the other end of the twisted string is fixed to the slider connected to the load through a fixture. An LVDT is installed at the load end to measure its displacement in real time; to prevent sudden movement of the load, the twisted string is pre-twisted by half a turn. During the experiment, the motor is controlled to rotate a specific number of times at a preset speed. Its rotation causes the two twisted strings to twist and contract, thereby making the load perform linear motion under the constraint of the linear guideway. Throughout the process, the LVDT accurately records the changes in load displacement and transmits the data to a computer for analysis. An analysis of the recorded displacement data can yield displacement characteristics under different load and twisted string material conditions. The key geometric parameters are set as follows: the initial string length is 15 cm, and the distance between the two strings is 1.4 cm.

To investigate the generality of variable-load TSA displacement characteristics across different string conditions, three distinct string materials were employed: ultra-high-molecular-weight polyethylene string, aramid string, and nylon string. Each string was tested with two diameters: 0.5 mm and 1 mm. Table 1 details the specific classification of each string type and its corresponding elastic moduli. These material and dimensional variations in strings were examined to study their impact on TSA performance.

### 2.2. Displacement Test

**Figure 3:**
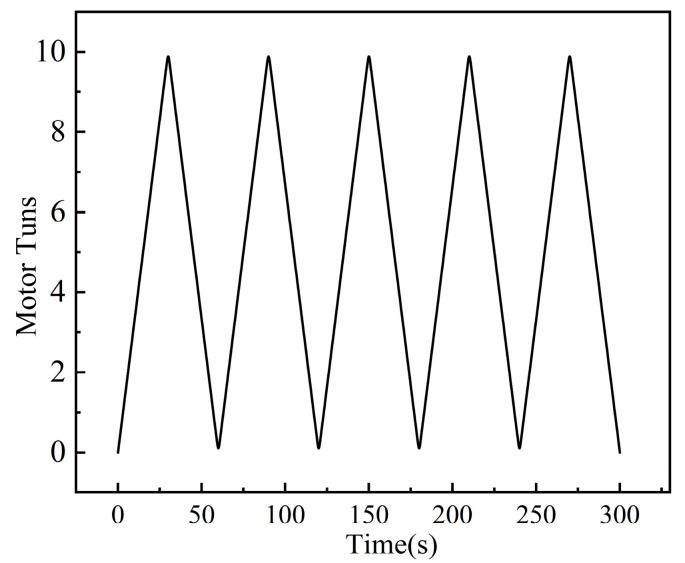
A typical test history of motor rotation turns with time.

Repeated experiments were conducted for each sample string in Table 1 under distinct loads (0.1 kg, 1 kg, 2 kg, 3 kg, 4 kg) using the established experimental platform. As shown in Figure 3, the procedure involved the following: To avoid a sudden contraction of the strings, which could cause jerky motion of the load, the strings are pre-twisted half a turn, followed by 10 full clockwise rotations, immediately succeeded by 10 counter-clockwise rotations. Under different load conditions, the same motor execution program is run cyclically. Throughout this process, the displacement sensor recorded end-effector displacement data at 0.5 s intervals. Material-specific datasets under corresponding load conditions were transmitted to a computer for analysis.

**Figure 4:**
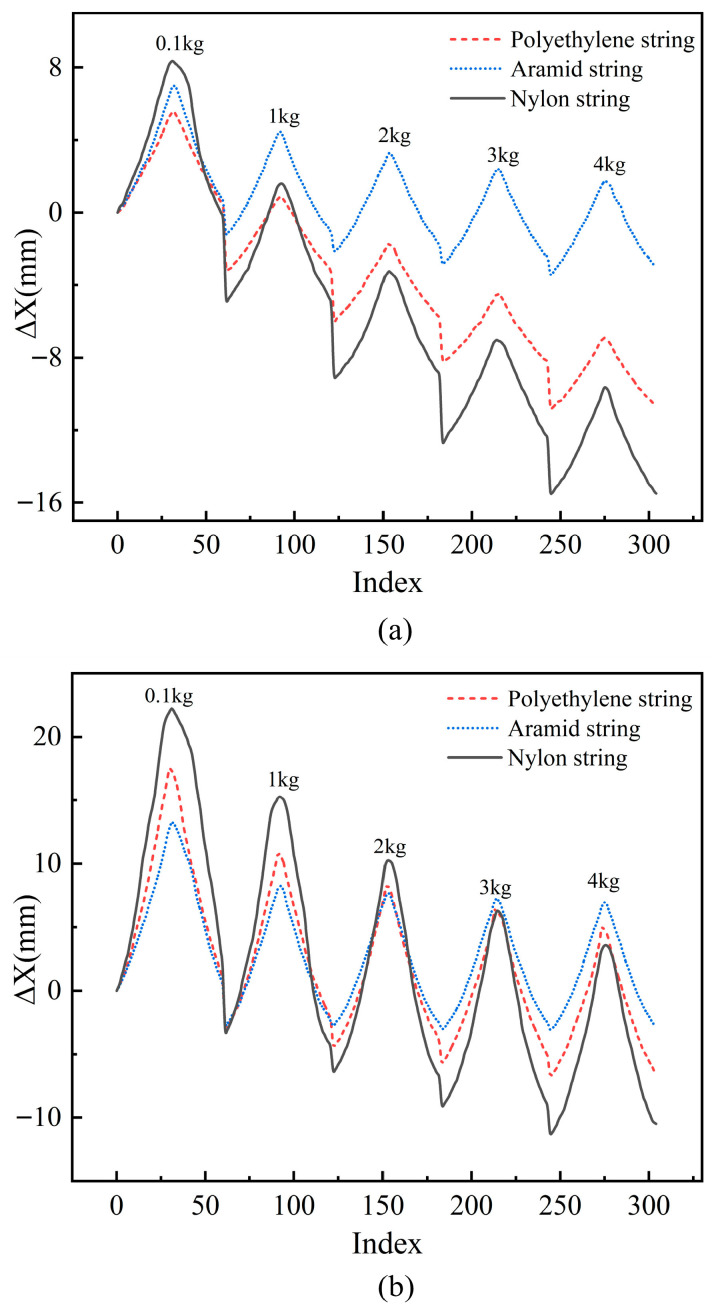
Experimental results of displacement Δ*X* versus sample point indexes for three material strings (polyethylene string, aramid string, and nylon string) with diameters of 0.5 mm (**a**) and 1 mm (**b**) under loads of 0.1 kg, 1 kg, 2 kg, 3 kg, and 4 kg, respectively.

Experimental results are presented in Figure 4. Figure 4a displays displacement profiles of 0.5 mm diameter polyethylene, aramid, and nylon strings under five distinct loads (0.1 kg, 1 kg, 2 kg, 3 kg, 4 kg), respectively; Figure 4b shows corresponding displacement distributions for 1 mm diameter strings (the x-axis “Index” represents sampling point sequence, while the y-axis “Δ*X*” denotes TSA-driven displacement).

To analyze the displacement variation trend of the TSA under different load conditions and explore the impact of load changes on its displacement performance, five groups of load conditions with significant intervals (0.1 kg, 1 kg, 2 kg, 3 kg, 4 kg) were selected for the experiment. Under each load condition, the displacement of the TSA at 0.1 kg was taken as the initial position, which served as the baseline for analysis against subsequent displacement data. The experimental results reveal significant changes in the driving displacement under different load conditions. Specifically, as the load increases, both the initial position and maximum displacement of the TSA show a decreasing trend. Additionally, the results indicate the presence of obvious nonlinear factors in the displacement variation. This phenomenon highlights the load as a key factor influencing the displacement performance and driving capacity of the TSA, with its nonlinear characteristics posing new challenges for system design and control. Therefore, to improve the accuracy of displacement prediction, it is essential to thoroughly consider load factors in the modeling process and enhance the model’s description of nonlinear behavior in order to minimize the impact of load variations on displacement estimation.

## 3. Mechanical Approach

### 3.1. Variable-Load Mechanical Approach

In this section, drawing on the research findings of Singh et al. [17], we employ mechanical analysis methods to examine the TSA with offset double-twisted strings. Additionally, we perform a comparative experimental analysis to systematically explore variable load conditions, aiming to derive more accurate conclusions.

**Figure 5:**
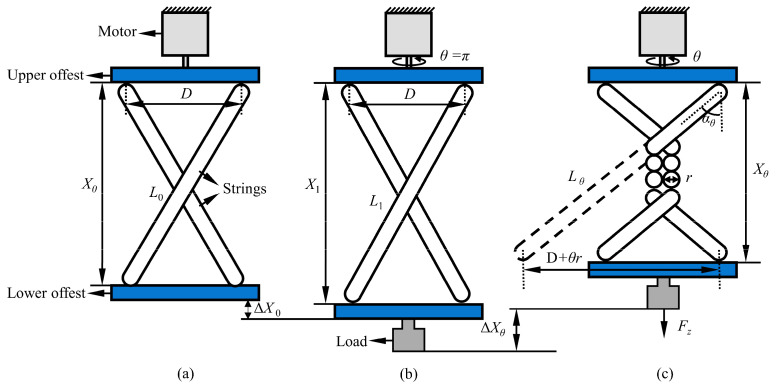
The schematic diagram of TSA’s mechanical analysis approach. (**a**) The initial state for a pre-twisted half turn without load. (**b**) The state for a pre-twisted half turn with load. (**c**) The state with load after the motor makes a certain number of turns *θ*.

During our analytical approach, we assume that the radius of the twisted string remains unchanged during the twisting process, unaffected by tangential compressive deformations, and that the stiffness of the twisted string remains constant. Based on these assumptions, we derive the relationship between the driving displacement and the input. As shown in Figure 5, the simplified structure of the TSA consists of a motor, two twisted strings, and a suspended load. Specifically, the upper end of the twisted strings is connected to the driving motor through the upper offset module, while the lower end is connected to the load through the lower offset module.

To prevent sudden motion of the load due to the twisted string’s abrupt contraction, as illustrated in Figure 5a, we initialize the system at the state when the motor completes half a turn. The twisted string’s initial length is $L_{0}$ without load, and the offset between the strings is D. The initial distance between the upper and lower offset modules of the TSA can be calculated, denoted as $X_{0}$, with its calculation formula given below:

(1) $$X_{0}=\sqrt{{L_{0}}^{2}-D^{2}}$$

When a working load $F_{z}$ is applied to the lower end of the TSA (as shown in Figure 5b), the twisted string is subjected to tension, causing its length to change from $L_{0}$ to $L_{1}$. Correspondingly, the distance between the upper and lower offset modules changes from $X_{0}$ to $X_{1}$, which can be expressed as follows:

(2) $$X_{1}=\sqrt{L_{1}-D^{2}}$$

(3) $$L_{1}=L_{0}+\frac{F_{z}}{K}\cdot \frac{X_{0}}{L_{0}}$$

In the formula, K is the stiffness coefficient of the twisted string. Then, as the motor rotates through an angle θ, the strings twist accordingly, forming a cylindrical shape with a radius of r at their midsection, as illustrated in Figure 5c.

This leads to a further increase in the tension experienced by the strings. Consequently, the length of the twisted string changes from $L_{1}$ to $L_{\theta }$. The distance between the upper and lower offset modules changes from $X_{1}$ to $X_{\theta }$, which in turn drives the load to produce linear motion, with the displacement denoted as $\Delta X_{\theta }$. $L_{\theta }$ can be calculated by

(4) $$L_{\theta }=L_{0}+\frac{F_{\theta }}{K}$$

(5) $$F_{\theta }=\frac{F_{Z}}{2\cos\alpha_{\theta }}$$

In the formula, $F_{\theta }$ denotes the axial tension when the twisted string is twisted by an angle of θ, and $F_{Z}$ represents the load force. In addition, $\alpha_{\theta }$ is the angle between the twisted string and the direction of the motor shaft when the string is twisted by an angle of θ. We assume that the helix angle $\alpha_{\theta }$ of the cylinder formed by the middle part of the twisted string is uniformly distributed over the entire cylindrical surface; thus, this helix angle is equal to the angle $\alpha_{\theta }$ between the twisted string and the direction of the motor shaft, and $\cos\alpha_{\theta }$ can be expressed by

(6) $$\cos\alpha_{\theta }=\frac{X_{\theta }}{\sqrt{{X_{\theta }}^{2}+\left(D+\theta r\right)}}$$

(7) $$X_{\theta }=L_{\theta }\cos\alpha_{\theta }=L_{0}\cos\alpha_{\theta }+\frac{F_{Z}}{2K}$$

Therefore, the displacement of the load driven by the TSA can be expressed as

(8) $$\Delta X_{\theta }=X_{\theta }-X_{1}=L_{0}\cos\alpha_{\theta }+\frac{F_{Z}}{2K}-X_{1}$$

### 3.2. Analysis of Mechanical Approach

**Figure 6:**
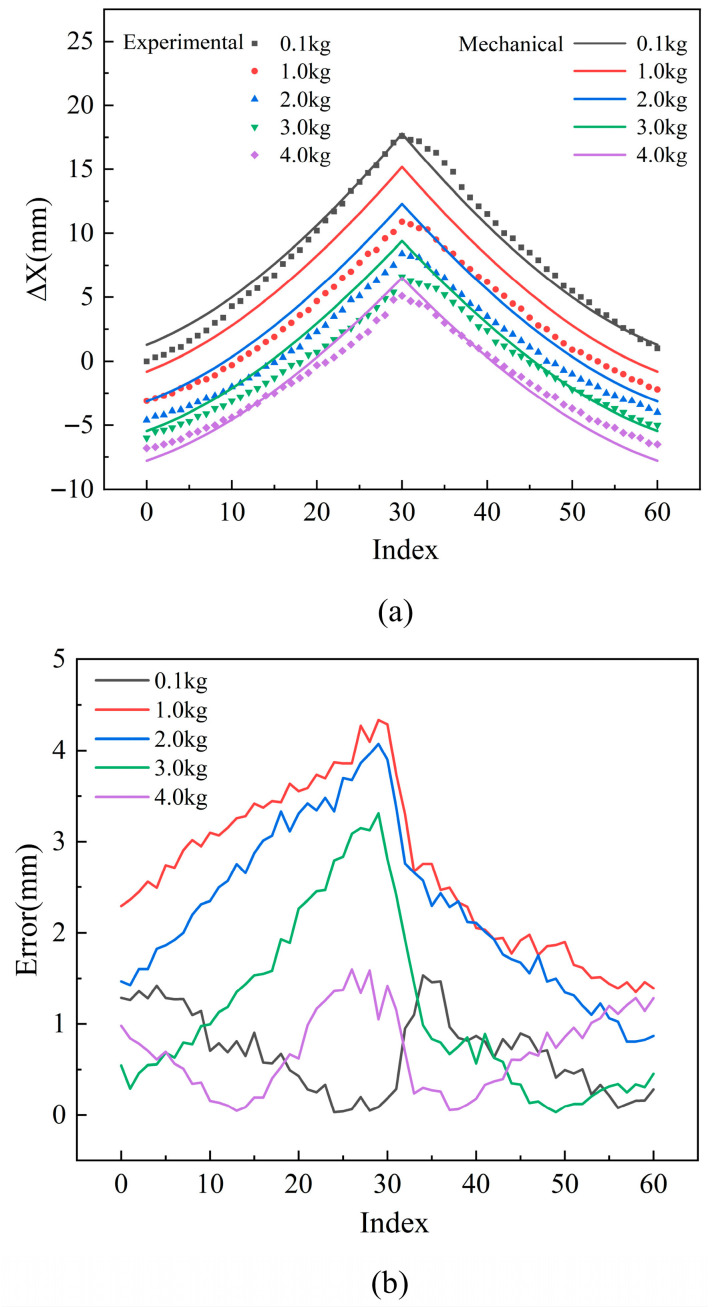
Comparison between the TSA with polyethylene string of 1 mm mechanical approach predictions and experimental results under different loads (0.1 kg, 1 kg, 2 kg, 3 kg, and 4 kg): (**a**) displacement Δ*X* (**b**) error.

To validate the accuracy of the aforementioned mechanical analysis approach, we designed and conducted a series of comparative experiments. In these experiments, polyethylene strings with a diameter of 1 mm were tested under various loading conditions (0.1 kg, 1 kg, 2 kg, 3 kg, and 4 kg). Using the mechanical approach mentioned above, the corresponding displacements under each load condition were calculated. Figure 6 presents a comparison between the experimental data and the mechanical approach predictions.

Figure 6a compares the experimental displacements of the 1 mm polyethylene string under different load conditions with the theoretical predictions obtained from the traditional mechanical approach. In the figure, the scatter points represent the experimental data, while the solid lines indicate the theoretical results. The analysis reveals that the traditional mechanical approach exhibits noticeable deviations in predicting TSA displacements. Under low loads (e.g., 0.1 kg), the predicted values closely match the experimental results; however, as the load increases, the discrepancy between the two becomes more pronounced. Interestingly, when the load reaches higher values (e.g., 4 kg), the deviation tends to decrease again, indicating a lack of robustness in the approach’s predictive stability across different load ranges. Figure 6b illustrates the differences between the experimental and calculated values, showing a maximum error of up to 4 mm. Considering the relatively small displacement range of the TSA and the high precision required in control applications, such a level of prediction error is insufficient to meet the performance requirements for accurate actuator modeling.

## 4. BPNN Model

When conducting theoretical modeling of the TSA, we made a series of assumptions to simplify the derivation and intentionally ignored some influencing factors. As a result, the traditional variable-load model’s displacement prediction accuracy under different load conditions was limited. To overcome this deficiency, this paper proposes a variable-load model based on the BPNN. The BPNN can adapt to complex input–output relationships and significantly reduce the reliance on prior assumptions [28,29]. We trained the network using a comprehensive dataset covering a wide range of operating conditions, aiming to provide more accurate performance predictions for the TSA in variable-load environments. This method significantly improves the accuracy of displacement prediction by explicitly introducing key factors that the traditional model failed to consider, providing more reliable theoretical support for the design and control of TSA.

### 4.1. Model Establishment

**Figure 7:**
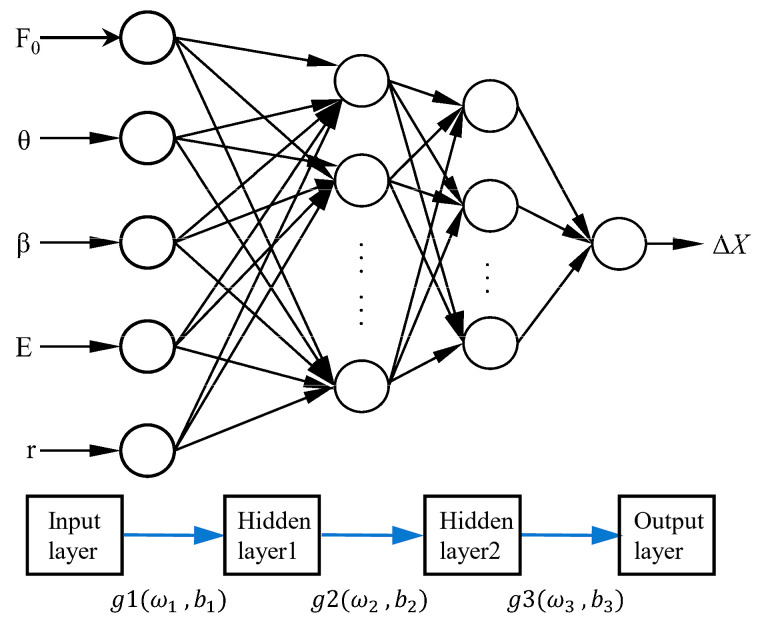
Schematic of the BPNN model structure.

The BPNN has significant advantages in the field of complex system modeling due to its powerful nonlinear mapping ability and multi-layer fully connected structure. Its supervised learning mechanism iteratively adjusts network weights through the error back propagation algorithm, effectively minimizing the difference between predicted output and the true value. In this study, we constructed a BPNN model based on the key parameters for displacement calculation of the TSA, and trained and tested it using experimental data. Figure 7 shows the proposed double-hidden-layer BPNN structure for establishing the displacement response model of TSAs under different loading conditions. Its core framework is as follows:

(1) Input–Output Architecture

Based on theoretical analysis, the variation in TSA displacement is influenced by multiple factors, including load magnitude, twisted angle, twisted direction, the twisted string’s Young modulus, and the string’s diameter. To comprehensively account for these factors, we incorporate them as input feature vectors for the BPNN. The output layer of the network consists of a single node that represents the change in TSA displacement.

(2) Hidden Layer Design

The number of neurons in the hidden layer is determined by the complexity of the problem, the size of the input/output layers, and the desired error tolerance. Using an empirical formula as a reference, we selected a dual-hidden-layer neural network model with a 10×8 configuration. Through sensitivity analysis, the optimal number of nodes was determined, creating a four-layer neural network structure with 5 × 10 × 8 × 1.

(3) Training Strategy

**Figure 8:**
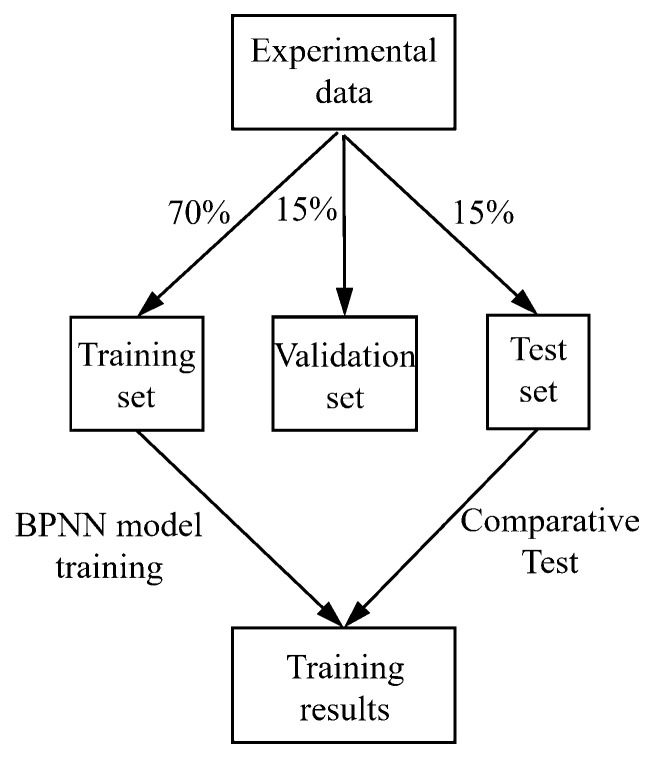
Validation process of the BPNN model.

Figure 8 illustrates the training and testing process using the BPNN. The dataset used for model training includes 150 independent experimental tests, covering all combinations of the parameters to be studied (including three materials, two radii, five loads, with five repeated experiments for each combination). Each experiment generates 60 data points, resulting in a total of 9000 data points. The dataset was partitioned by test cycles using stratified sampling into a training set (70%), a validation set (15%), and a test set (15%)—this was performed to avoid data leakage and ensure that each subset has representative distribution characteristics. In terms of data preprocessing, the study adopted a detailed workflow: First, numerical encoding was applied to the twisting direction; Second, the core elastic modulus (E) of the string was used to characterize material properties, instead of using categorical labels; Additionally, all input features underwent min–max normalization based on parameters calculated from the training set, scaling the data to the interval [0, 1], whereas no scaling was applied to the output displacement. During the training process, the MATLAB 2023aNeural Network Toolbox was used, with standardized training sample data as input. To enhance training efficiency and stability, the hyperbolic tangent function (tansig) was chosen as the activation function for the hidden layer, while the linear function (purelin) was used for the output layer. The training function employed was traingdx, and the performance function was Mean Squared Error (MSE). The training process was set to a maximum of 5000 iterations, with an early stopping mechanism: if the validation error did not decrease for 10 consecutive iterations, training was automatically halted. Additionally, the initial learning rate was set to 0.01, with a 5% decay applied every 100 iterations.

### 4.2. Analysis of Model Results

**Figure 9:**
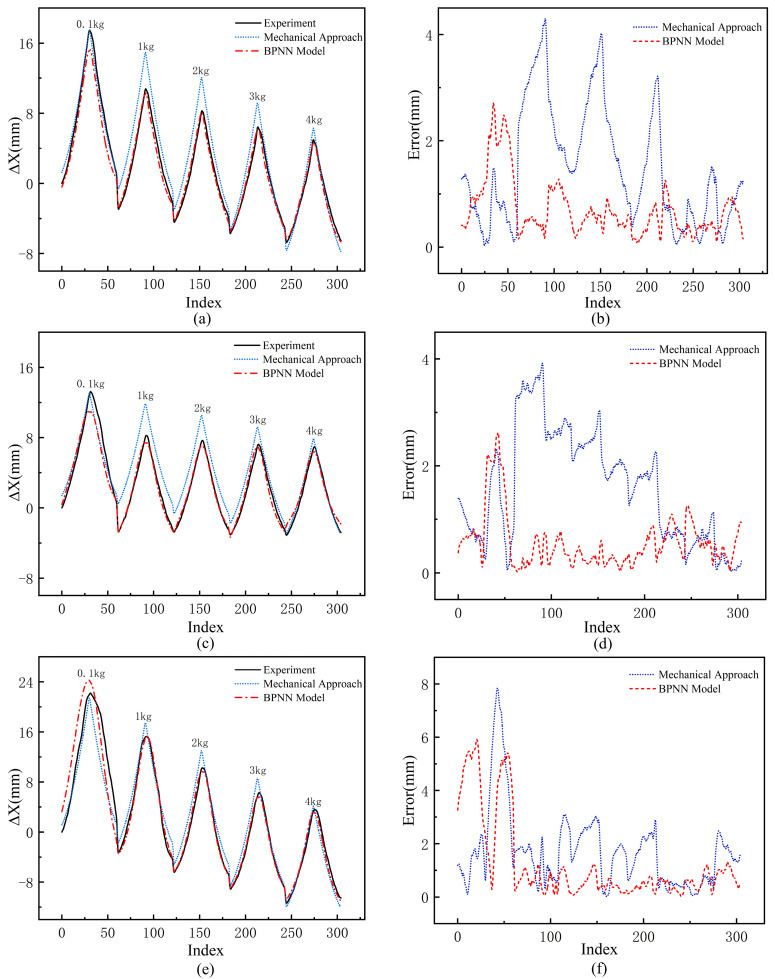
Comparison of BPNN model, mechanical approach, and experimental values for three materials with 1 mm diameter, respectively: displacement Δ*X* for polyethylene string (**a**), aramid string (**c**), and nylon string (**e**); error for polyethylene string (**b**), aramid string (**d**), and nylon string (**f**).

To validate the performance of the proposed BPNN model in modeling the TSA under variable load conditions, we selected three different materials for the twisted strings, all with a diameter of 1 mm: polyethylene string, aramid string, and nylon string. Figure 9a,c,e present a comparison of the BPNN model’s prediction results with those of the traditional mechanical approach and experimental measurements under different material conditions. In the figures, the red dashed line represents the BPNN model, with its predictions closely matching the experimental data; the blue dashed line represents the predictions of the traditional mechanical approach. Moreover, it is evident that the traditional approach is consistent with the experimental data only under low-load conditions (0.1 kg), while as the load increases, the deviation between the traditional approach’s predictions and the experimental values becomes significantly larger.

Figure 9b,d,f show the error comparisons between the BPNN model, the traditional mechanical approach, and the experimental measurements under different material conditions. The comparison results indicate that the traditional approach has significantly larger prediction errors. For polyethylene string and aramid string, the maximum error exceeds 25% under 1 kg and 2 kg loads. For nylon string, the maximum error exceeds 30% under a 0.1 kg load. In contrast, the BPNN model exhibits lower prediction errors overall, with most errors kept within 5%. Although its performance is slightly worse under the 0.1 kg low-load condition, its maximum error still does not exceed 20%.

The above analysis demonstrates the effectiveness of the BPNN model in TSA modeling under variable loads. By comparing it with the traditional mechanical approach and experimental measurements, the BPNN model aligns more closely with the experimental data, showing higher accuracy and lower errors. Compared to the traditional approach, the BPNN model improves accuracy by 50%. This approach exhibits stable performance across different materials and diameters, further confirming its universality and reliability in TSA displacement prediction.

To quantitatively evaluate the prediction performance of the proposed BPNN model, we conducted a comprehensive statistical analysis and compared it with the traditional mechanical model as well as two modern machine learning methods (Support Vector Regression (SVR) and Random Forest (RF)). In the analysis, we calculated key metrics including Mean Absolute Error (MAE), Mean Squared Error (MSE), Root Mean Squared Error (RMSE), and Coefficient of Determination (R^2^). The results (as shown in Table 2) indicate that the BPNN model not only significantly outperforms the traditional mechanical method but also exhibits superior accuracy and robustness over SVR and RF across all evaluation metrics. This demonstrates that the BPNN model can effectively capture the complex nonlinear relationships between TSA displacement and multiple influencing factors (e.g., load, motor rotation angle, string properties). Furthermore, the BPNN achieves a good balance between model complexity and interpretability, making it particularly suitable for engineering application scenarios that have high requirements for performance and practical deployment.

The development of a high-precision TSA model lays the foundation for the design of a dexterous hand-driven system. The next section will focus on the development of an anthropomorphic dexterous hand based on this model.

## 5. TSA-Driven Anthropomorphic Dexterous Hand

The TSA-driven anthropomorphic dexterous hand is characterized by its lightweight design and excellent compliance, fully leveraging the advantages of TSAs. In the previous sections, we significantly improved the prediction accuracy of the end displacement of the TSA-driven system through the established BPNN model, providing a solid theoretical foundation for the design of a TSA-driven dexterous hand. Building upon this prediction model, we will now further explore the overall design, kinematic analysis, and performance validation of the dexterous hand to ensure the high efficiency and reliability of the drive system in practical applications.

### 5.1. Design of the Dexterous Hand

The hand, one of the most delicate tools in the human body, has evolved over a long period of time, not only displaying unique esthetic beauty in its shape but also demonstrating exceptional diversity and adaptability in its structure and function. From grasping objects to expressing emotions, the hand’s multifunctionality makes it a key medium for human interaction with the world.

**Figure 10:**
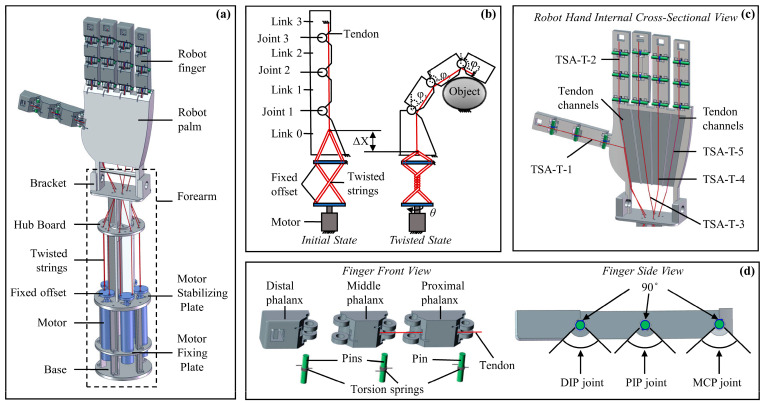
Overall assembly and structural details of the TSA-driven dexterous hand. (**a**) Overall assembly diagram of the dexterous hand. (**b**) Structure of a single finger and gripping mechanism. (**c**) Distribution of the tendons corresponding to the twisted strings inside the palm. (**d**) Exploded view of a single finger and schematic of joint stroke angles.

This section explores the construction, structural dimensions, and palm characteristic parameters of the human hand from a biomimetic perspective. Based on this, a novel flexible bionic dexterous hand has been designed and fabricated, as shown in Figure 10. Figure 10a presents the overall assembly drawing of the designed dexterous hand, which consists of a hand structure with five fingers and a palm, five TSAs, and a forearm. Each finger has three joints, with torsion springs equipped at the joints to provide rebound force. Both the fingers and the palm adopt a hollow structure design to reserve space for tendon-driven transmission. Five tendons are, respectively, connected to the fingertips of each finger, with the other end of each tendon linked to the corresponding TSA. The bending sequence of each phalanx is determined by the preset resistance gradient of the joint torsion springs. Since a single tendon drives a finger with three rotational degrees of freedom, the system exhibits underactuated characteristics. Except for the necessary joint connectors and palm bottom fixtures, which are connected by screws and nuts, the main structure of the dexterous hand is formed using 3D-printed resin, greatly reducing the overall weight. The final anthropomorphic dexterous hand (excluding the motor) weighs 335 g, with a size approximately 1.2 times that of a real human hand.

Figure 10b illustrates the working principle of a single finger. The designed TSA-driven dexterous hand’s finger consists of four linkages (Linkage 0, Linkage 1, Linkage 2, and Linkage 3), three rotational joints (Joint 1, Joint 2, and Joint 3), three torsion springs, one tendon, and the TSA system. The TSA system includes a motor, two twisted strings, and two fixed offsets. In this design, Linkage 0 is fixed, while Linkages 1–3 rotate around Joints 1–3, respectively, in response to the displacement of the TSA, thereby enabling the bending motion of the finger and completing gestures and grasping tasks. All three joints are equipped with torsion springs to provide the necessary elastic restoring force. Through this drive structure, the finger can perform flexible movements and adapt to various task requirements.

Figure 10c shows a cross-sectional view of the dexterous hand’s palm, clearly illustrating the internal tendon distribution of the palm and fingers. Five independently driven TSA units control each finger, with their movements being independent and non-interfering, ensuring good independence and coordination. By adjusting the motor’s rotation direction and the number of rotations, the corresponding tendon is stretched and relaxed, thereby driving the fingers to flex or extend.

Figure 10d presents an exploded view of a single finger, detailing its internal structure. The finger is composed of a proximal phalanx, middle phalanx, and distal phalanx connected in sequence. Torsion springs are integrated into the rotational joints between each phalanx to provide the necessary restoring force. The proximal phalanx is connected to the palm via a fixed linkage. The fingers passively return to their initial extended position via torsion springs integrated in all finger joints (MCP, PIP, and DIP). During the finger bending phase (when driven by the TSA), these springs are loaded; when the TSA untwists, the tendon tension is released, and the torsion springs provide the necessary restoring force, enabling the fingers to extend automatically. To enable human-like functional manipulation, each finger joint of the dexterous hand allows an adjustable range of motion from 0° to 90°.

The dexterous hand is driven by five independent TSAs (Twisted String Actuators). The TSAs themselves possess excellent compliance, and both the twisted strings and the internal tendons within the palm exhibit a certain degree of elasticity—this allows them to generate slight untwisting behavior when subjected to excessive external loads. This characteristic helps absorb impact energy and limits the maximum force transmitted from the actuators to the fingers and the target object, thereby providing a certain level of built-in safety protection. Furthermore, the dexterous hand adopts a structural design that combines joint springs with underactuated tendon transmission. By adjusting the torque of the torsion springs at each joint, the fingers can achieve natural bending along the intended motion trajectory. When grasping an object, this structure enables each finger segment to passively conform to the geometric shape of the object’s surface, without relying on complex real-time control systems or a large number of tactile sensors. We will verify these performances in subsequent experiments.

### 5.2. Kinematics Analysis

In this section, the kinematics of the dexterous hand are analyzed to derive the relationship between joint bending angles and TSA displacement. By coupling this model with the previously developed BPNN-based displacement prediction model, a complete control framework is established—from motor rotation angle to finger bending angles—laying a theoretical foundation for the precise control of TSA-driven dexterous hands.

**Figure 11:**
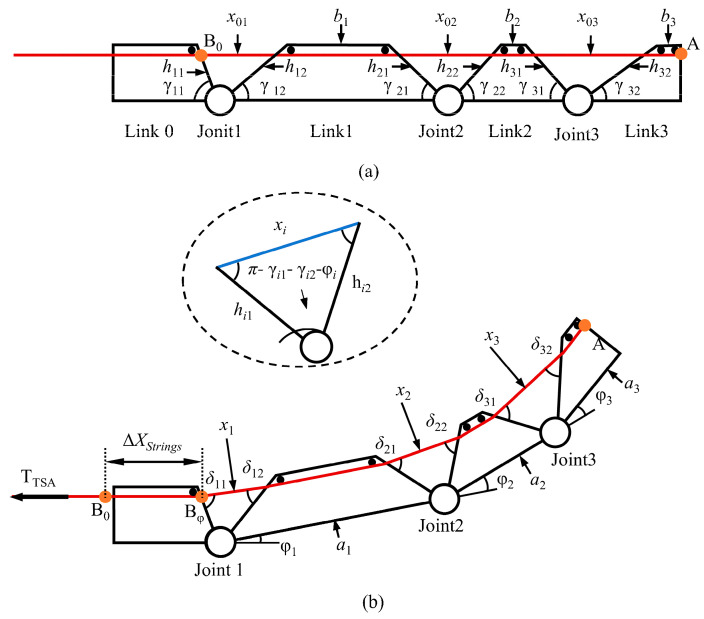
The schematic of single-finger movement: (**a**) initial state; (**b**) state after the finger is bent to a certain angle.

Given the similar motion and force characteristics across all fingers of the designed TSA-driven dexterous hand, a single finger (the middle finger) is selected for kinematic analysis. Figure 11 illustrates the motion of an individual finger. As shown in the initial state in Figure 11a, the length of the internal tendon is geometrically determined by the posture of the finger. The initial tendon length $L_{AB_{0}}$ can be calculated as follows:

(9) $$L_{AB_{0}}=\sum_{i=1}^{3}x_{0i}+\sum_{k=1}^{3}b_{k}$$

In the equation, $x_{0i}$ represents the tendon length corresponding to each joint, and $b_{k}$ denotes the length of the tendon within each link. Using the cosine law of triangles, the following relationship can be derived:

(10) $$x_{0i}=\sqrt{h_{i1}^{2}+h_{i2}^{2}+2h_{i1}h_{i2}\cos\left(\gamma_{i1}+\gamma_{i2}\right)}$$

In this context, $h_{i1}$ and $h_{i2}$ represent the sides of the triangle formed by the adjacent connecting links and the tendon. $\gamma_{i1}$ and $\gamma_{i2}$ are the angles of the links at the joint.

After the motor rotates by an angle θ, link 1, link 2, and link 3 of the finger rotate by angles *φ*1, *φ*2, and *φ*3 around Joints 1, 2, and 3, respectively, as shown in Figure 11b. Since the dimensions of each link remain constant, the length of the tendons inside the finger also remains unchanged. Therefore, the length of the tendon $L_{AB_{\varphi }}$ inside the finger can be calculated as

(11) $$L_{AB_{\varphi }}=\sum_{j=1}^{3}x_{j}+\sum_{k=1}^{3}b_{k}$$

In the equation, $x_{j}$ represents the tendon length corresponding to each joint after the motor rotates by an angle *θ*. Similarly, using the law of cosines, the tendon length can be obtained as

(12) $$x_{j}=\sqrt{h_{j1}^{2}+h_{j2}^{2}+2h_{j1}h_{j2}\cos\left(\gamma_{j1}+\gamma_{j2}+\varphi_{j}\right)}$$

In the equation, $\varphi_{j}$ represents the angle between the j-th finger segment and the horizontal direction. Therefore, the variation in the tendon length inside the finger can be calculated as

(13) $$\begin{array}{ll} \Delta X_{\mathrm{Strings}} & =L_{AB_{0}}-L_{AB_{\varphi }}=\Delta x_{1}+\Delta x_{2}+\Delta x_{3}\\ & =\sum_{i=1}^{3}\sqrt{h_{i1}^{2}+h_{i2}^{2}+2h_{i1}h_{i2}\cos\left(\gamma_{i1}+\gamma_{i2}\right)}\\ & -\sum_{j=1}^{3}\sqrt{h_{j1}^{2}+h_{j2}^{2}+2h_{j1}h_{j2}\cos\left(\gamma_{j1}+\gamma_{j2}+\varphi_{j}\right)} \end{array}$$

In the equation, $\Delta x_{1}=x_{0i}-x_{i}i=1,2,3$.

Additionally, since the tendon is connected to the twisted strings at the end of the TSA, the change in length of the tendon inside the finger is equal to the displacement of the TSA. Thus, through the above kinematic model, we can clearly describe the relationship between the bending angle of the dexterous hand’s fingers and the displacement of the twisted strings.

### 5.3. Validation of Joint Angle Prediction Model

This paper combines the relationship model between the twisted strings’ displacement and the motor rotation angle established by the BPNN and the model of the twisted strings displacement and the finger bending angle derived based on kinematic theory to construct a prediction model of the motor rotation angle–finger bending angle. To verify the accuracy of this fusion model, we conducted an open-loop control experiment of the dexterous hand.

**Figure 12:**
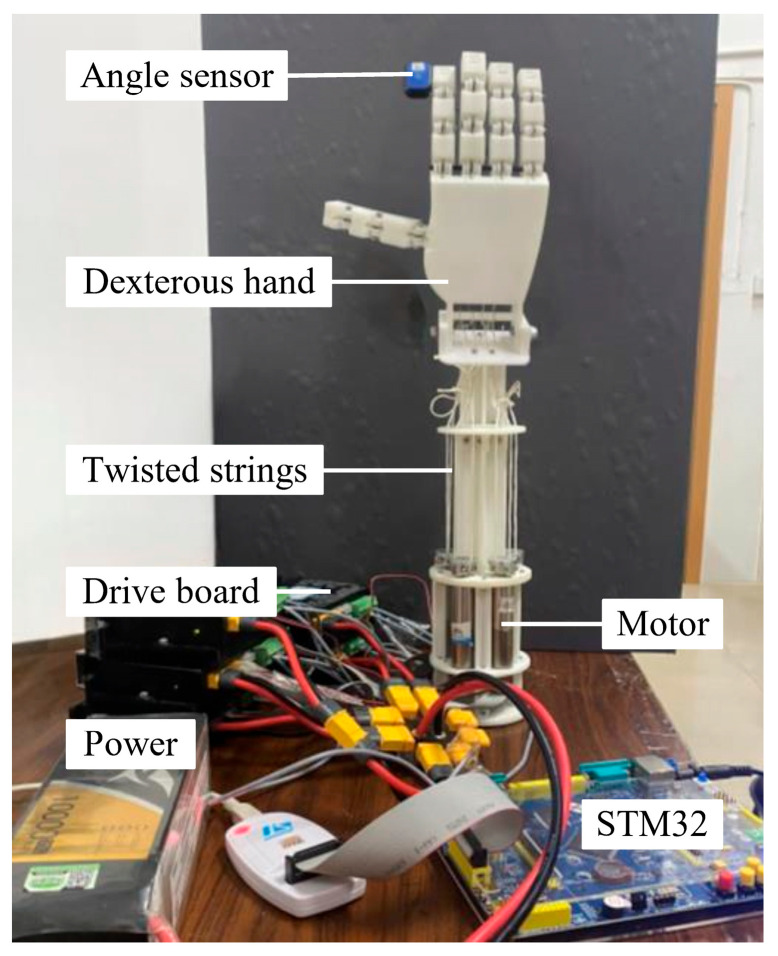
Experimental setup to evaluate the dexterous robotic hand.

As shown in Figure 12, the experimental setup includes the dexterous hand, Maxon DCX16L motor (integrated with Maxon ENX16 encoder), RMDS-405 motor driver, STM32 microcontroller, and inertial measurement unit (IMU, WY9011DCL-BT50). The motor is powered by a 24 V DC power supply and controlled by the driver. In the experiment, 1 mm aramid string was chosen as the twisted string, and the index finger was selected as the test object because the five fingers of the dexterous hand have a similar structure. The experiment was conducted under constant temperature (23 ± 1 °C) and humidity (40% RH) conditions to eliminate the influence of temperature drift on the elastic modulus of the twisted string. During the experiment, the motor was controlled to drive the twisted string to fully bend the finger and then return to the original position. The angle sensor recorded the finger bending angle and synchronized it to the computer. The focus was on measuring the bending angle of the index finger’s fingertip. Additionally, the number of motor twists was also recorded, and these data were correlated with time to determine the relationship between the motor twist and the finger bending angle. The motor operated at a constant speed of 40 rpm ± 5%, and the twist number was set in steps of 0.5 turns to unify the dynamic characteristics. Data were collected after each step, maintaining a steady state for 200 ms. The experiment was repeated three times.

**Figure 13:**
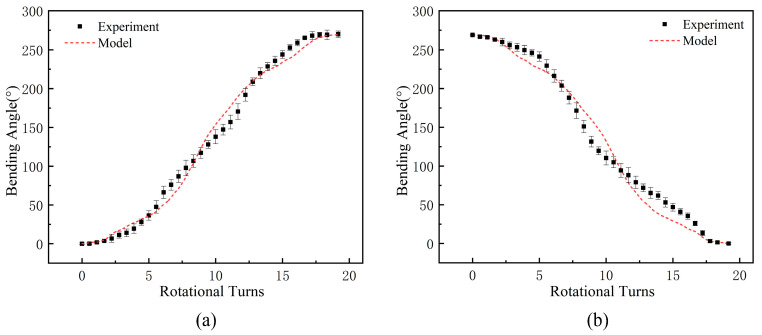
Comparison between the actual bending angle trajectories of the index finger’s fingertip and the bending angle trajectories predicted by the established fusion model: (**a**) flexion process; (**b**) extension process.

The collected experimental results were compared with the prediction results of the model, combining the BPNN model of the TSA and the kinematic model of the dexterous hand. The comparison results are shown in Figure 13. Figure 13a,b present the comparison of the experimental values and the model prediction values of the bending angle for the index finger’s fingertip angle during the finger flexion and extension process. The black dots represent the average values of the three experiments, and the error bars show the experimental errors. The red dashed line represents the prediction results of the fusion model of the BPNN and kinematic theory. The results indicate that the established model can accurately capture the dynamic change trend of the joint angle. Analyzing the data in the figure, it can be seen that in the initial and final stages of the string twisting, the model prediction results are basically consistent with the experimental results, with an error of no more than 2°, which effectively ensures the accuracy of the initial positioning of the dexterous hand and the stability of the final grasping force. In the middle stage of the twist, due to the combined influence of friction between the string and the internal guide holes of the dexterous hand, hysteresis effect, and other uncertain factors, the experimental data exhibit a certain degree of dispersion, posing a challenge to the model prediction. However, the prediction errors of the models are all below 20%, corresponding to an absolute error of less than 15°, which meets the basic accuracy requirements for the control of dexterous hand joints. The experimental verification of the above prediction models provides a theoretical guarantee for the practical application of dexterous hands. In the next section, systematic performance tests will be conducted on dexterous hands.

## 6. Experiments and Analysis on the Comprehensive Performance of Dexterous Hands

The core performance indicators of the dexterous hand lie in its ability for flexible and coordinated movement of the five fingers, which directly determines its potential for simulating human hand actions, grasping diverse objects, and performing complex operations. To comprehensively evaluate the overall performance of the designed TSA-driven anthropomorphic dexterous hand, this section systematically conducted experiments in four aspects: human-like gesture tests, multi-target grasping tests, human–robot interaction tests, and mechanical performance tests.

### 6.1. Human-like Gesture Test

**Figure 14:**
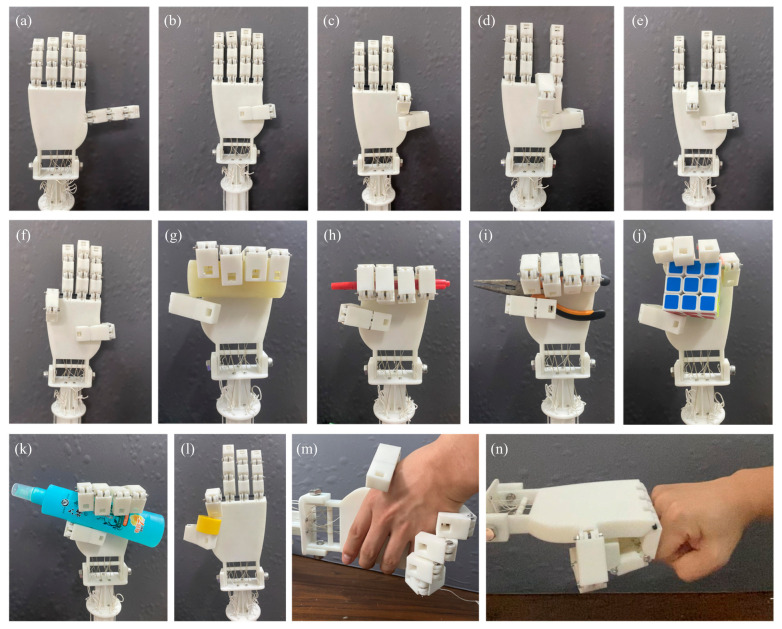
Performance testing of TSA-driven dexterous hand: (**a**–**f**) human-like gesture tests; (**g**–**l**) multi-target grasping tests; (**m**,**n**) human–robot interaction tests.

First, we evaluated the dexterous hand’s ability to simulate typical human hand postures. The five fingers are driven by five motors, which are controlled by five RMDS-405 drivers and one STM32 controller. By precisely controlling each TSA unit and adjusting finger postures—with specified motor rotation counts and speeds—each finger moves along a preset trajectory to complete designated gestures. These include full extension, independent thumb bending, and sequential thumb contact with the index, middle, ring, and little fingers (as shown in Figure 14a–f). The experimental results show that the dexterous hand can effectively reproduce the precise pinching actions of the thumb, index finger, and middle finger. However, the dexterous hand encounters limitations in enabling the thumb to fully reach the distal ends of the ring and little fingers. This is primarily due to two factors: the restricted length of the thumb joints and its current design, which lacks the rotational freedom at the thumb base characteristic of the human hand. Specifically, it is difficult for the thumb to fully bend to the distal phalanx of the little finger while the little finger remains extended. Future work can enhance its human-like flexibility by introducing rotational freedom at the base of the thumb.

### 6.2. Multi-Object Grasping Test

The practical grasping ability of the dexterous hand was further evaluated. The test was considered successful if the object was actively grasped and stably held for at least 5 s. First, the TSA dexterous hand was adjusted to the initial position with all five fingers open. Subsequently, the object was placed within the operating range of the dexterous hand, and the grasping action was performed under specific motor settings, referring to common human grasping strategies. Two grasping strategies were mainly verified: palm wrapping and fingertip pinching (as shown in Figure 14g–l). Under the palm wrapping strategy, it not only successfully grasped slender objects such as pens but also smoothly grabbed larger items like Rubik’s cubes. This demonstrates good coordination between the fingers and the palm, as well as a strong wrapping force exerted by the palm. Figure 14l shows the fingertip pinching test of the dexterous hand, and its success in grasping small cylindrical blocks confirms its precise grasping ability. In the grasping experiments, the dexterous hand successfully grasped objects of varying shapes, sizes, and weights, including daily items (e.g., cylinders, floral water bottles, Rubik’s cubes) and specific tools (e.g., pliers). This demonstrates that when handling objects of different sizes, the inherent compliance of the TSAs and the passive adaptability provided by the joint torsion springs and tendons together endow the dexterous hand with excellent compliant characteristics.

However, it should be noted that the lack of adduction–abduction degrees of freedom in the dexterous hand’s metacarpophalangeal (MCP) joints restricts its ability to efficiently enclose larger or irregularly shaped objects.

### 6.3. Human–Robot Interaction Test

To explore the application potential of the dexterous hand in physical human–robot interaction (HRI), two typical interaction scenarios were demonstrated. In Figure 14m, the dexterous hand mimicked human hand postures and completed a handshake with the operator. In Figure 14n, the dexterous hand extended its palm, stably resisted externally applied thrust, and maintained its posture. These demonstrations preliminarily verified the feasibility and compliance of the TSA-driven dexterous hand in physical interaction tasks, laying a foundation for human–robot interaction applications.

### 6.4. Mechanical Performance Test

To evaluate the mechanical output capability of the dexterous hand, two tests were conducted: the grasping force test and fingertip force test.

**Figure 15:**
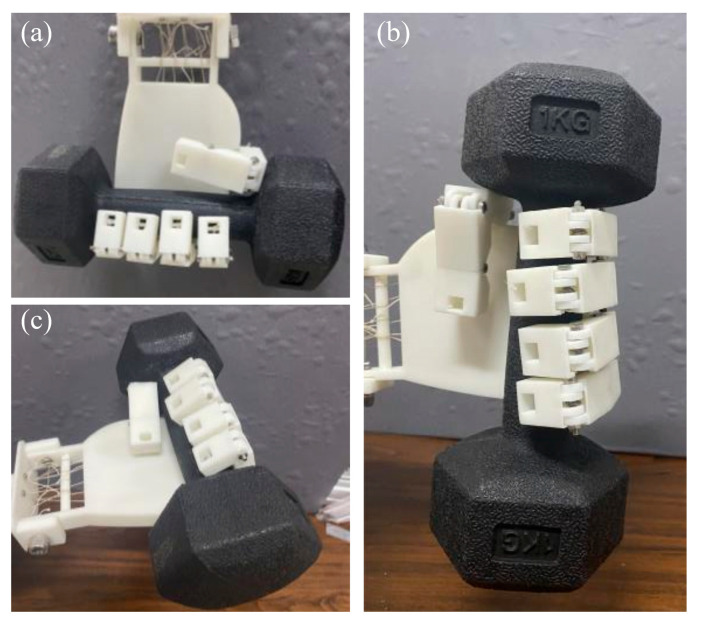
Grasping force test of TSA-driven dexterous hand: (**a**) vertical grasp; (**b**) horizontal grasp; (**c**) lateral grasp.

The grasping force test includes three directional grasps: vertical grasp, horizontal grasp, and lateral grasp, as shown in Figure 15. The vertical grasp involves placing the dexterous hand vertically upside down, positioning the dumbbell within its grasping range, and then driving it to bend all five fingers to grip the dumbbell tightly. The horizontal grasp entails placing the dexterous hand with its palm facing upward and driving its fingers to bend and grasp the dumbbell. The lateral grasp refers to positioning the dexterous hand with its palm facing sideways, then activating its fingers to bend and grip the dumbbell. The test was completed by gradually increasing the weight of the dumbbell. Experimental results showed that the dexterous hand could stably grasp and lift a dumbbell with a weight of 1 kg, indicating that its maximum grasping force is ≥10 N, which can meet the grasping requirements of most lightweight objects.

**Figure 16:**
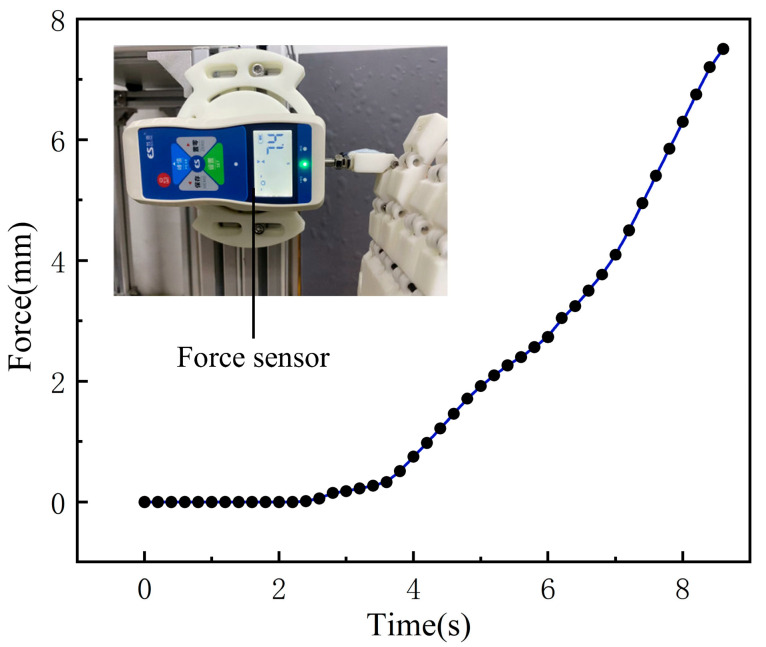
TSA-driven dexterous fingertip force testing.

The fingertip force test further evaluated the dexterous hand’s fine manipulation ability, using a pressure sensor to measure the output force of the fingertip. As shown in Figure 16, the pressure sensor was fixed on a rigid base (aluminum profile), and the lower end of the dexterous hand was fixed. The position was adjusted so that a single finger (e.g., the index finger) could press the sensing unit of the sensor vertically and frontally during the bending process. Driven by the motor, the finger was continuously bent and pressed, with pressure data recorded in real time. Test results showed that the maximum contact force at the fingertip can reach 7.4 N, providing the necessary force output guarantee for fine operations.

The comprehensive test results above indicate that the dexterous hand designed in this study achieves an excellent balance between a lightweight structure and high force output. The total weight of this dexterous hand is only 335 g (excluding motors), which is significantly lighter compared with complex multi-actuator systems such as the DEXMART Hand [30]. Meanwhile, its maximum fingertip force reaches 7.4 Newtons, enabling it to stably complete a variety of grasping and interaction tasks. Although lighter dexterous hands like the UC Soft Hand are even lighter (280 g), they only use three actuators to drive five fingers, which is far inferior in terms of flexibility and independent finger control capability to the five independent TSAs adopted in this study [31]. On the other hand, high-force-output dexterous hands, such as the dual-mode dexterous hand developed by Team Kim (with a maximum fingertip force of 31.3 N) [32], need to rely on more complex and heavier drive mechanisms to achieve high performance. In summary, the dexterous hand proposed in this study gains significant advantages in the balance between light weight, output force, structural simplicity, and inherent compliance, providing a competitive design solution for safe and adaptive human–robot interaction applications.

## 7. Conclusions and Future Work

This study addresses the challenges of strong nonlinearity and load sensitivity in TSAs for anthropomorphic dexterous hands through an integrated approach of data-driven modeling and biomimetic mechanism innovation. The main contributions are summarized as follows:

(1) A data-driven modeling method utilizing a dual hidden-layer BPNN was developed to predict TSA displacement under variable loads (0.1–4.2 kg). This method significantly improved accuracy compared to traditional mechanical models, with most errors within 5% across different string materials and diameters.
(2) A lightweight, underactuated anthropomorphic dexterous hand was developed, featuring a biomimetic three-phalanx structure, tendon-spring transmission, and 3D-printed resin construction. Weighing 335 g (excluding motors) and sized 1.2 times a human hand, it integrates the BPNN model with kinematic analysis to accurately map motor rotation to finger bending angles.
(3) An experimental validation confirmed the system’s performance: it replicates typical human hand gestures, stably grasps diverse objects with a maximum fingertip force of 7.4 N, and exhibits compliance in human–robot interaction.

This work advances TSA modeling for variable-load scenarios and presents a framework for designing lightweight, high-performance dexterous hands, with potential applications in service robotics, rehabilitation, and precision assembly. However, the current model does not account for hysteresis and nonlinear dynamic effects, remains sensitive to environmental variations, and operates under open-loop control without real-time feedback. Future efforts will focus on three main directions: (1) enhancing the TSA model to incorporate hysteresis, dynamic effects, and environmental influence, enabling online adaptation to time-varying conditions; (2) improving the hand’s mechanical design, including adding thumb base rotation and MCP joint abduction–adduction, to enhance grasping versatility; and (3) implementing closed-loop control with embedded sensors to achieve robust manipulation under external disturbances in unstructured environments.

## Figures and Tables

**Table 1:** Parameters of various sample strings.

NO.	Material	Diameter	Young’s Modulus
1	Polyethylene string	1 mm	14 GPa
2		0.5 mm	
3	Aramid string	1 mm	100 GPa
4		0.5 mm	
5	Nylon string	1 mm	1.4 GPa
6		0.5 mm	

**Table 2:** Performance comparison of predictive models across evaluation metrics.

Model	MAE	MSE	RMSE	R^2^
Mechanical	1.603	3.670	1.918	0.782
BPNN	0.578	0.601	0.775	0.921
SVR	0.847	1.215	1.102	0.874
RF	0.793	1.086	1.042	0.887

## Data Availability

The data and code presented in this study are available as follows:The source code and implementation details are openly available in the GitHub repository at: https://github.com/bailixi2123/TSA_BPNN.git (accessed on 6 August 2025). The complete dataset is available upon request from the corresponding author due to project-specific restrictions.
